# Hyperphosphatemic Familial Tumoral Calcinosis With a Large Hip Mass

**DOI:** 10.7759/cureus.81756

**Published:** 2025-04-05

**Authors:** Issa Ali, Yehuda Galili, Teresa Bernardes, Steve Carlan

**Affiliations:** 1 Department of Internal Medicine, Cleveland Clinic Florida, Weston, USA; 2 Department of Oncology and Hematology, Cleveland Clinic Florida, Weston, USA; 3 Department of Medicine, Orlando Regional Medical Center, Orlando, USA; 4 Department of Academic Affairs and Research, Orlando Regional Medical Center, Orlando, USA

**Keywords:** autosomal recessive disease, bone mass, diffuse soft tissue calcinosis, genetic syndromes, hyperphosphatemia

## Abstract

Hyperphosphatemic familial tumoral calcinosis (HFTC) is a rare autosomal recessive disorder that generally presents in the first two decades of life with ectopic calcification throughout the body. The underlying metabolic disorder is caused by a mutation in a gene responsible for regulating fibroblast growth factor 23 (FGF23) activity. Loss of regulation of FGF23 results in hyperphosphatemia resulting in the characteristic tissue calcinosis deposits, especially in periarticular locations. The diagnosis is made with imaging, hyperphosphatemia, and genetic testing. Medical and surgical treatments are recommended to reduce blood phosphate and remove the masses.

A 35-year-old male presented with a painful left lateral hip mass that had been gradually enlarging over the past four months. X-rays showed amorphous calcific densities in the left hip consistent with tumoral calcinosis. Magnetic resonance imaging (MRI) noted a stable complex mass in the left posterior hip and ischio-femoral space consistent with tumoral calcinosis. He had an elevated phosphorus level of 6.0 mg/dL (reference range 2.5 to 4.5 mg/dL). Excision of the mass was successful and genetic testing showed a pathogenic variant in polypeptide N-acetylgalactosaminyltransferase 3 (*GALNT3*), associated with HFTC. He was treated with diet and sevelamer (phosphate binder), and discharged.

This case demonstrates that the detection of the disorder can be delayed by the slow progression of clinical symptoms and tumor calcinosis over time. Ultimately, early awareness of the disease and the mechanisms responsible for the tissue damage are important to limit long-term health consequences, which can include calcifications that ulcerate and limit joint motion. As seen in this patient, the condition often requires repeated surgical interventions. Finally, this is an autosomal recessive inherited disorder, so genetic counseling is an important component of comprehensive care.

## Introduction

Hyperphosphatemic familial tumoral calcinosis (HFTC) is a rare autosomal recessive disorder caused by mutations in one of three genes: polypeptide N-acetylgalactosaminyltransferase 3 (*GALNT3*), fibroblast growth factor 23 (*FGF23*), and Klotho (*KL*) [1]. Each mutation impairs normal FGF23 activity, a key phosphate homeostasis regulator [2]. Impaired FGF23 activity leads to hyperphosphatemia due to increased renal tubular reabsorption of phosphate and reduced renal phosphate excretion. Hyperphosphatemia ultimately results in the deposition of calcium phosphate crystals in soft tissues (tumoral calcinosis) and painful bone swelling (hyperostosis) [3,4]. HFTC typically presents in childhood or adolescence, with calcifications commonly affecting the hips, shoulders, and elbows, leading to joint stiffness, inflammation, and skin ulceration [5]. It can also involve blood vessels or the brain, and dental abnormalities. Patients eventually become symptomatic as the masses ulcerate or interfere with joint movement [6]. The diagnosis is supported by characteristic imaging and laboratory findings, including hyperphosphatemia with increased renal tubular reabsorption of phosphate, elevated or inappropriately normal levels of 1,25-dihydroxyvitamin D3, and increased C-terminal FGF23 fragments. The renal tubular reabsorption test method requires a fasting serum and random urine sample, and the value is calculated based on phosphorus and creatinine levels. When clinical and laboratory findings are inconclusive, genetic testing can confirm the diagnosis by identifying pathogenic variants in one of the three identified mutated genes, *GALNT3*, *FGF23*, or *KL* [6]. HFTC exemplifies how molecular defects can present as complex systemic diseases, often leading to misdiagnosis as more common conditions such as osteomyelitis, autoimmune connective tissue disease, panniculitis, osteoarthritis, or tendinitis, which ultimately can delay appropriate management [7]. Understanding HFTC provides insight into phosphate regulation and ectopic calcification and highlights the importance of genetic testing and potential for targeted therapies in advancing patient care and outcomes.

## Case presentation

**Figure 1:**
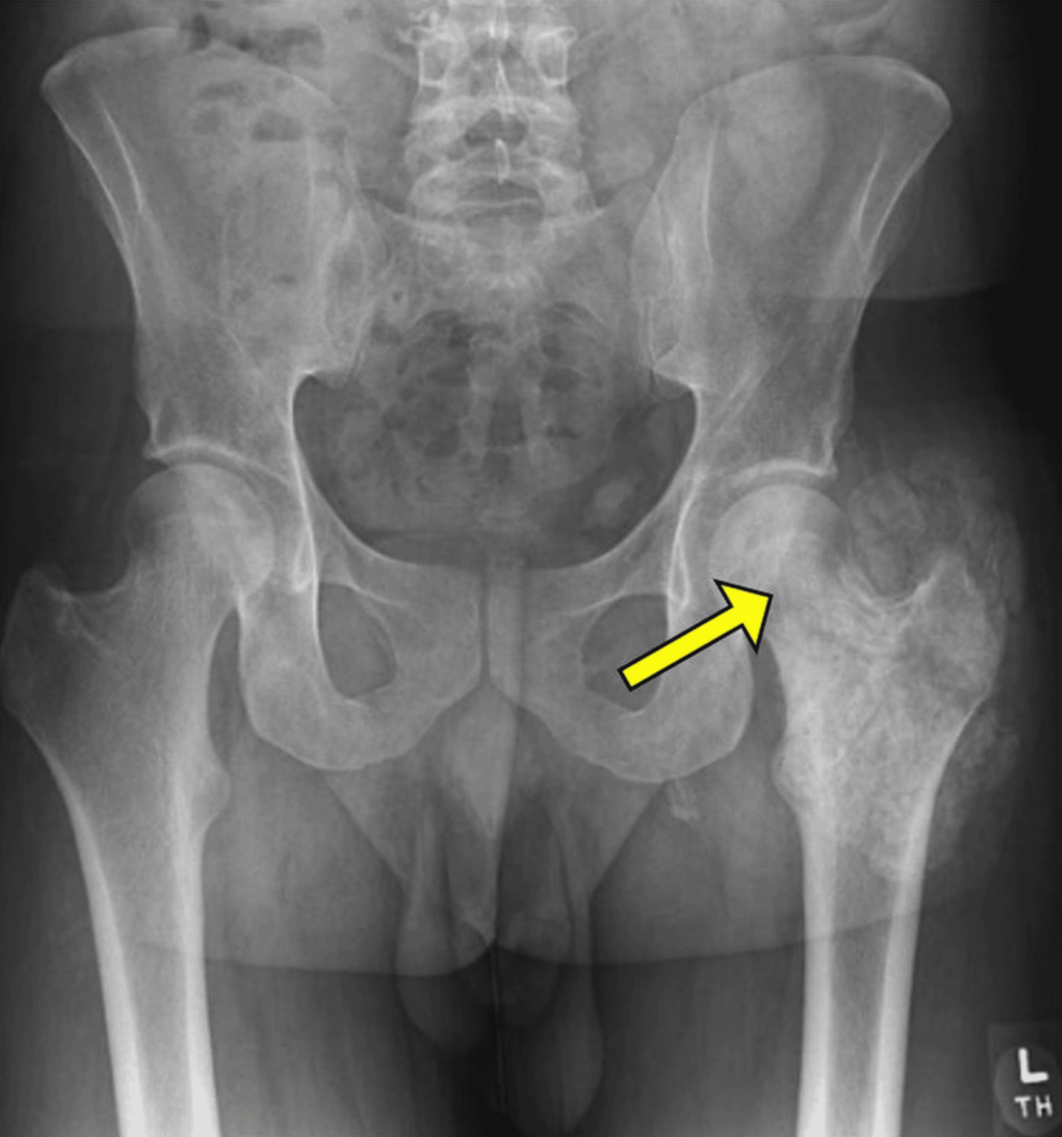
X-ray pelvis: amorphous calcific densities are shown by the yellow arrow

A 35-year-old male from abroad presented with a left lateral hip mass that had been gradually enlarging over the past four months. He had a history of right hip pain and a gluteal lump diagnosed seven years ago, which was excised. At that time, an MRI (magnetic resonance imaging) revealed calcification, and a biopsy confirmed tumoral calcinosis. Follow-up imaging showed no recurrence. The patient did not undergo laboratory testing to confirm an underlying diagnosis. Family history was unremarkable. Five years after the initial event, he developed bilateral iliotibial band (ITB) syndrome and calcific tendinitis. To treat the ITB syndrome, the patient had a series of four corticosteroid injections into the area of ITB inflammation over one year. The patient presented to outpatient primary care with complaints of a progressively enlarging mass in the left lateral hip, accompanied by intense pain that worsened with prolonged sitting/standing, lying on the affected side, activities of daily living, and recreational activities. Additionally, he experienced nocturnal pain in the left hip and intermittent left elbow pain. Vital signs were unremarkable. Physical examination revealed an ill-defined mass on the lateral aspect of the left hip. Laboratory findings were concerning for an elevated phosphorus level of 6.0 mg/dL (reference range 2.5 to 4.5 mg/dL), and other values were noncontributory, including a hemogram and metabolic panel. There was no evidence of connective tissue disease. No secondary causes, such as trauma, neoplasms, or infection, were identified. Initial x-rays were suggestive of a common extensor calcific tendinosis in the left elbow, and amorphous calcific densities in the left hip (Figure 1) were consistent with a history of tumoral calcinosis. 

**Figure 2:**
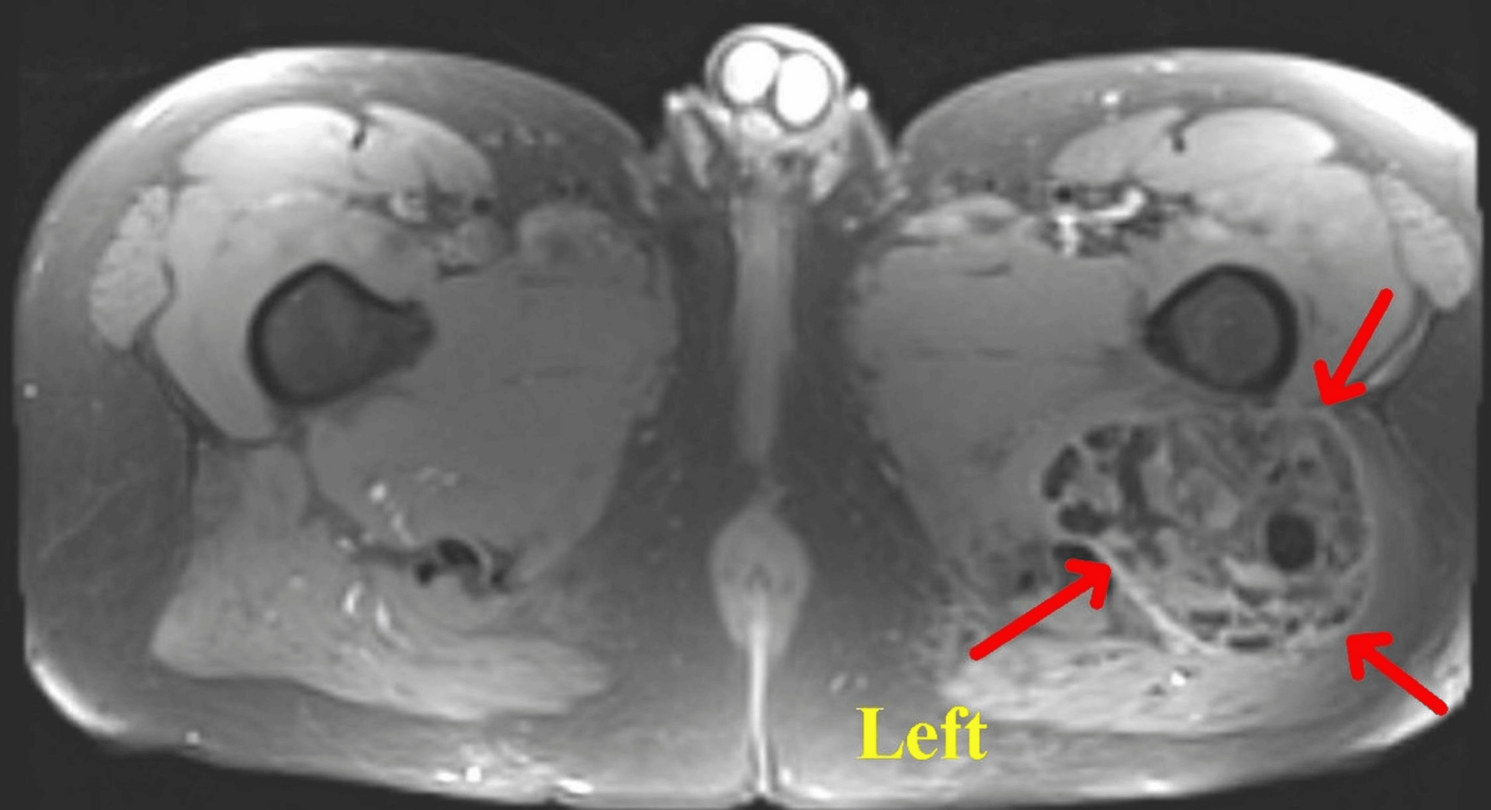
MRI hip cross section: Stable complex mass in the posterior left hip and ischio-femoral space with amorphous calcifications, likely tumoral calcinosis, and associated marrow edema in the proximal femur, possibly due to impingement from the mass The mass measures 15.7 x 9.0 x 9.2 cm. MRI, magnetic resonance imaging.

**Figure 3:**
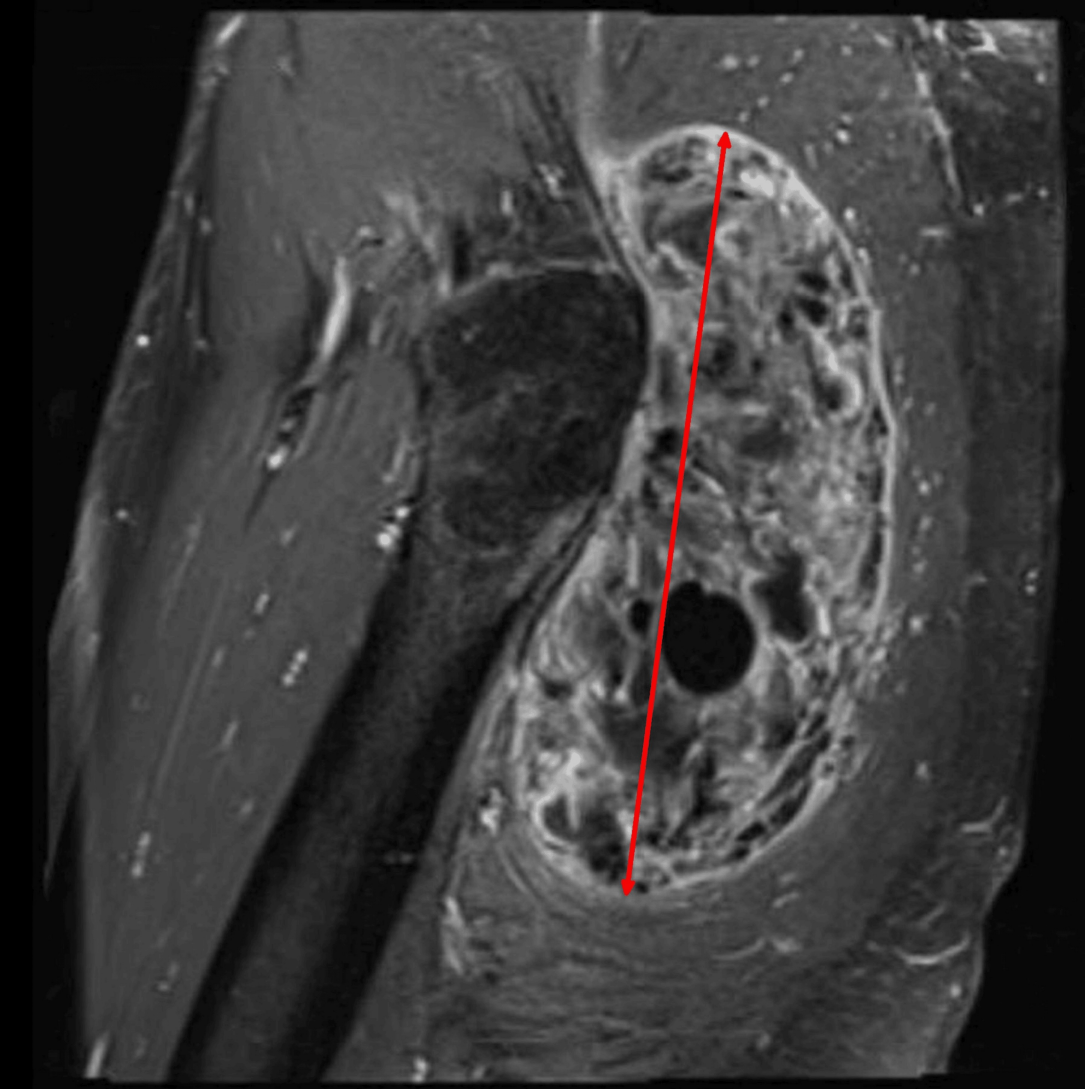
MRI hip: Sagittal view of the mass shown in Figure 2 The distance between the arrow heads is 15.7.

The patient was referred and evaluated by sports medicine and orthopedic surgery. A follow-up MRI noted a stable complex mass in the left posterior hip and ischio-femoral space with adjacent marrow edema suggesting impingement, consistent with tumoral calcinosis, and a left anterosuperior labral tear without high-grade cartilage wear (Figures 2, 3). 

The patient was deemed an appropriate candidate for surgery, and after informed consent, he received an excision of the mass. Pathology of the tissue confirmed tumoral calcinosis with multinucleated giant cell reaction, chronic inflammation, and fibrosis. On evaluation by nephrology, the patient was deemed to have tumoral calcinosis in the setting of normal renal function. Genetic testing showed a pathogenic variant in *GALNT3*, associated with HFTC. The patient’s genetic report noted that he was homozygous for an autosomal recessive mutation, causing a frameshift in exon 6 of the *GALNT3* gene. This variant is predicted to introduce a premature stop codon at least 50 nucleotides upstream of the canonical donor splice site of the penultimate exon, which results in the loss of function of the protein product due to nonsense-mediated mRNA decay. This mutation is the most common of the three types.

Expanded laboratory work was consistent with HFTC disease, noting an elevated phosphorus level of 6.0 mg/dL, a markedly elevated FGF23 level of 2,414 RU/mL (reference range <180 RU/mL for adults), a low 1-25 vitamin D level of 12 ng/mL (reference range for adults, 20 to 50 ng/mL), and a normal parathyroid hormone (PTH) level of 18 pg/mL (reference range of 10 to 65 pg/mL) (Table 1).

**Table 1:** Lab values

Lab value	Normal lab values	Day 1	Day 5
Serum phosphate (mg/dL)	2.5 to 4.5	6.0	6.0
Fibroblast growth factor 23 (FGF23) level (RU/mL)	<180 for adults		2414
1-25 Vitamin D level (ng/mL)	For adults, 20 to 50		12
Parathyroid hormone level (pg/mL)	10 to 65		18

The patient was started on sevelamer, advised to maintain a low-phosphorus diet, with a plan for vitamin D supplementation once phosphorus is better controlled. He was counseled about the implications of the condition and the genetic details and discharged to follow-up as an outpatient with dietary consultation. He was seen for a single outpatient visit before being lost to follow-up. His condition had improved.

## Discussion

The patient’s initial presentation with a progressively enlarging left hip mass, bilateral ITB syndrome, and calcific tendinitis reflects the systemic nature of HFTC [5]. These manifestations are often misdiagnosed as common orthopedic conditions such as tendinopathy or bursitis, contributing to diagnostic delays. The elevated phosphorus level (6.0 mg/dL) and imaging findings of calcific densities were key diagnostic clues. However, the diagnosis was ultimately confirmed by genetic sequencing from whole blood samples, which identified a pathogenic variant in *GALNT3*. The identification of elevated FGF23 levels and low 1,25-dihydroxyvitamin D confirmed the impaired phosphate handling typical of HFTC [8]. This case underscores the diagnostic complexity of HFTC due to its nonspecific musculoskeletal symptoms and the potential for misdiagnosis as other calcification disorders or isolated disease processes [7]. HFTC due to a *GALNT3* mutation results from a loss-of-function mutation in the *GALNT3* gene, which encodes the enzyme ppGalNAc-T3. This enzyme is essential for the O-glycosylation of FGF23. The mutation leads to increased FGF23 cleavage, reduced biologically active FGF23, impaired renal phosphate excretion, and hyperphosphatemia [3,4]. Elevated serum phosphate causes an elevated calcium-phosphate component and calcium-phosphate deposition in the skin and soft tissues, leading to painful masses, joint stiffness, and limited mobility [9-11]. Without diagnosis and management, the condition often requires repeated surgical interventions to address complications, as occurred in our patient. This autosomal recessive condition is considered genetically heterogeneous since three different mutant genes can disrupt normal FGF23 homeostasis using different molecular mechanisms. The most common mutation is the kind our patient suffered with, the *GALNT3* mutation. The second and third most common variants are the *FGF23* and *KL*, respectively [10]. Patients typically present for treatment when they become symptomatic and that reflects the area of the body involved. One of the largest masses removed was reported in a 15-year-old with a 1.2 kg calcified mass of the thigh measuring 20 cm x 15 cm x 10 cm [12]. The hip mass in our patient was also huge at 15.7 cm x 9.0 cm x 9.2 cm. Treatment includes non-steroidal anti-inflammatory drugs, corticosteroids, probenecid, dietary phosphate restriction, and phosphate binders (e.g., sevelamer) to lower serum phosphate. Interleukin-1 (IL-1) antagonists may manage inflammation, and experimental therapies targeting FGF23 signaling are under investigation [9,13]. Due to its rarity and phenotypic variability, the diagnosis relies on genetic testing, which is essential for guiding treatment. Future research should focus on targeted therapies to modulate FGF23 and anti-inflammatory agents to manage systemic symptoms better [9,11].

This case highlights the importance of early recognition and diagnosis of HFTC to prevent complications such as joint destruction, skin ulceration from calcified masses, and long-term disability. The impact on quality of life, including chronic pain and restricted mobility, underscores the need for a multidisciplinary approach involving nephrology, orthopedics, and rheumatology. Opportunities for future research should focus on improving the understanding of FGF23 signaling and developing more effective therapies to modulate phosphate homeostasis and reduce the inflammatory response associated with calcification [14]. Transmission of autosomal recessive conditions is predictable, and therefore, once the disorder is confirmed, the patient should receive detailed genetic counseling.

## Conclusions

HFTC is a rare yet significant disorder caused by one of three mutations leading to impaired FGF23 activity and subsequent hyperphosphatemia. This case reinforces the value of a comprehensive metabolic workup in patients with recurrent calcifications and musculoskeletal complaints. Utilizing multi-specialty healthcare provider options can be helpful in cases where a diagnosis is undetermined. Timely diagnosis and management of HFTC through phosphate-lowering strategies and targeted surgical intervention are essential to improving patient outcomes and reducing disease burden. Despite current treatment limitations, ongoing research into targeted therapies and FGF23 modulation offers hope for improved patient outcomes. Genetic counseling of patients with this autosomal recessive disorder is an essential component of their care.

Despite current treatment limitations, ongoing research into targeted therapies and FGF23 modulation offers hope for improved patient outcomes. Considering the inheritance pattern of autosomal recessive disease, genetic counseling of patients is an essential component of their care.

## References

[1] Dayal D, Gupta S, Kumar R, Srinivasan R, Lorenz-Depiereux B, Strom TM (2021). A novel homozygous variant in exon 10 of the GALNT3 gene causing hyperphosphatemic familial tumoral calcinosis in a family from North India. Intractable Rare Dis Res.

[2] Yu X, White KE (2005). FGF23 and disorders of phosphate homeostasis. Cytokine Growth Factor Rev.

[3] Garringer HJ, Fisher C, Larsson TE (2006). The role of mutant UDP-N-acetyl-alpha-D-galactosamine-polypeptide N-acetylgalactosaminyltransferase 3 in regulating serum intact fibroblast growth factor 23 and matrix extracellular phosphoglycoprotein in heritable tumoral calcinosis. J Clin Endocrinol Metab.

[4] Kato K, Jeanneau C, Tarp MA (2006). Polypeptide GalNAc-transferase T3 and familial tumoral calcinosis. Secretion of fibroblast growth factor 23 requires O-glycosylation. J Biol Chem.

[5] Boyce AM, Lee AE, Roszko KL, Gafni RI (2020). Hyperphosphatemic tumoral calcinosis: Pathogenesis, clinical presentation, and challenges in management. Front Endocrinol (Lausanne).

[6] Ramnitz MS, Gafni RI, Collins MT (2018). Hyperphosphatemic familial tumoral calcinosis. GeneReviews® [Internet].

[7] Olsen KM, Chew FS (2006). Tumoral calcinosis: Pearls, polemics, and alternative possibilities. Radiographics.

[8] Slavin RE, Wen J, Kumar D, Evans EB (1993). Familial tumoral calcinosis. A clinical, histopathologic, and ultrastructural study with an analysis of its calcifying process and pathogenesis. Am J Surg Pathol.

[9] Ramnitz MS, Gourh P, Goldbach-Mansky R (2016). Phenotypic and genotypic characterization and treatment of a cohort with familial tumoral calcinosis/hyperostosis-hyperphosphatemia syndrome. J Bone Miner Res.

[10] Kaszycki M, Villalpando B, Hickson L (2024). Hyperphosphatemic familial tumoral calcinosis. South Med J.

[11] Dumitrescu CE, Kelly MH, Khosravi A (2009). A case of familial tumoral calcinosis/hyperostosis-hyperphosphatemia syndrome due to a compound heterozygous mutation in GALNT3 demonstrating new phenotypic features. Osteoporos Int.

[12] Claramunt-Taberner D, Bertholet-Thomas A, Carlier MC, Dijoud F, Chotel F, Silve C, Bacchetta J (2018). Hyperphosphatemic tumoral calcinosis caused by FGF23 compound heterozygous mutations: What are the therapeutic options for a better control of phosphatemia?. Pediatr Nephrol.

[13] Ichikawa S, Austin AM, Gray AK, Allen MR, Econs MJ (2011). Dietary phosphate restriction normalizes biochemical and skeletal abnormalities in a murine model of tumoral calcinosis. Endocrinology.

[14] Imel EA, Econs MJ (2005). Fibroblast growth factor 23: Roles in health and disease. J Am Soc Nephrol.

